# Supplementary figures and images for: Abnormal mechanical stress on bicuspid aortic valve induces valvular calcification and inhibits Notch1/NICD/Runx2 signal
Source: PeerJ. 2023 Mar 6;11:e14950. doi: 10.7717/peerj.14950 (PMC9997191; doi:10.7717/peerj.14950)

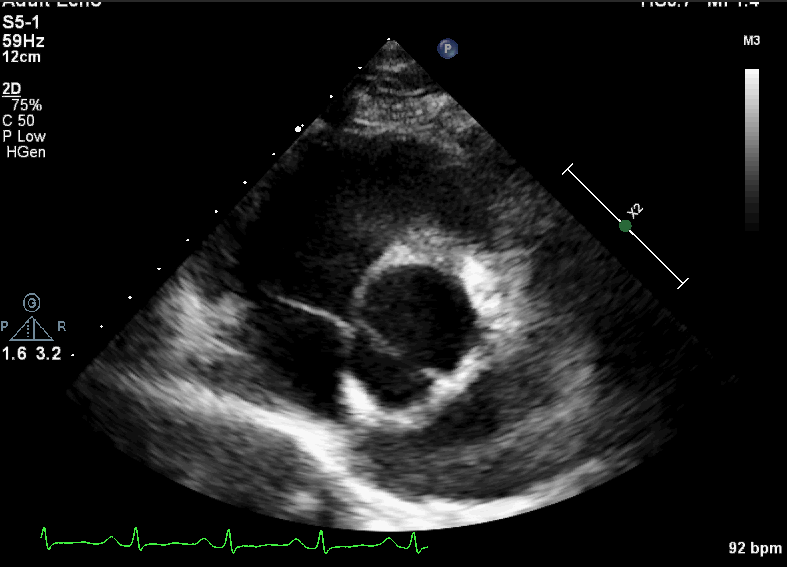

Supplement: Data S1 [file peerj-11-14950-s001.zip › Figure 1/BAV1.png]

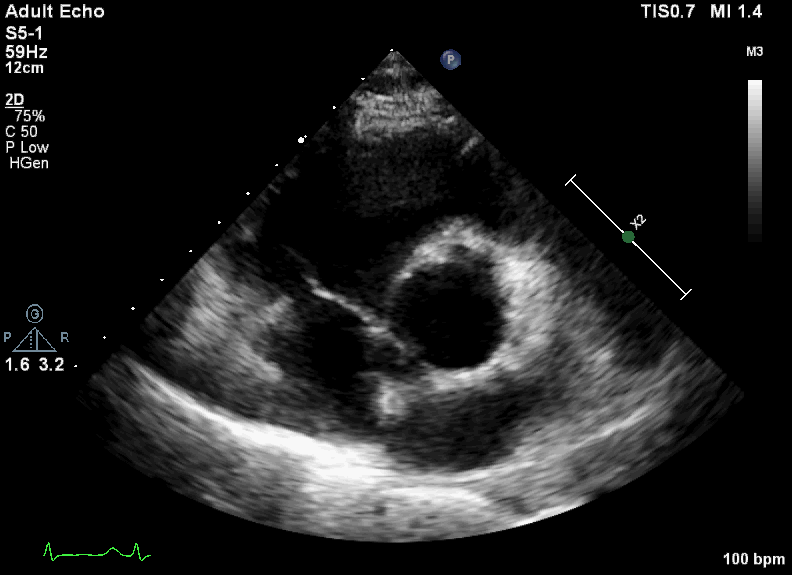

Supplement: Data S1 [file peerj-11-14950-s001.zip › Figure 1/BAV2.png]

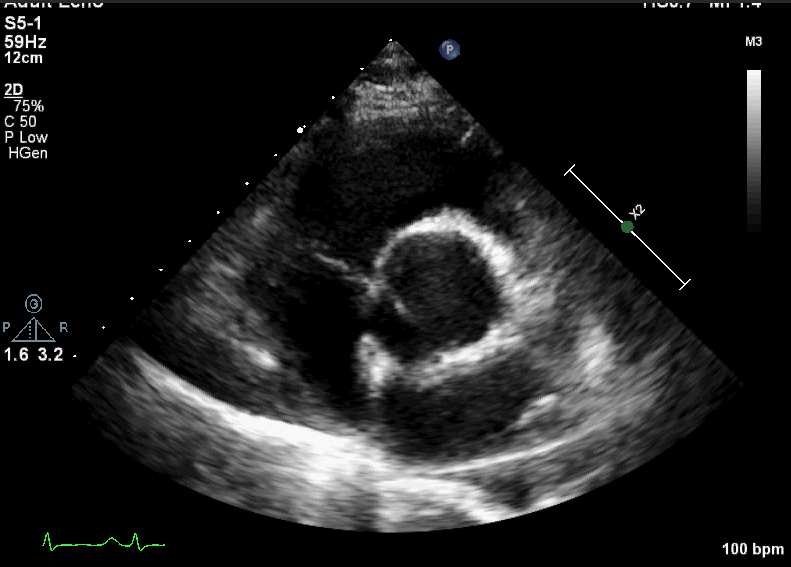

Supplement: Data S1 [file peerj-11-14950-s001.zip › Figure 1/BAV3.png]

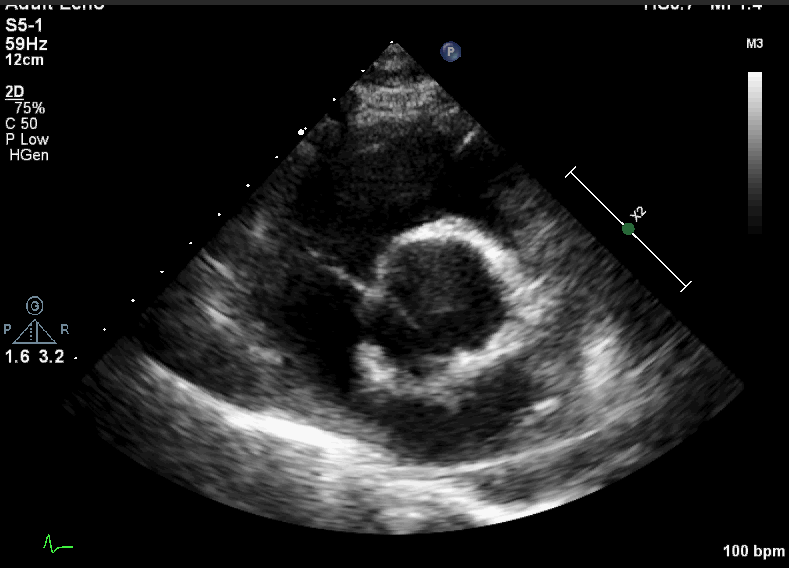

Supplement: Data S1 [file peerj-11-14950-s001.zip › Figure 1/BAV4.png]

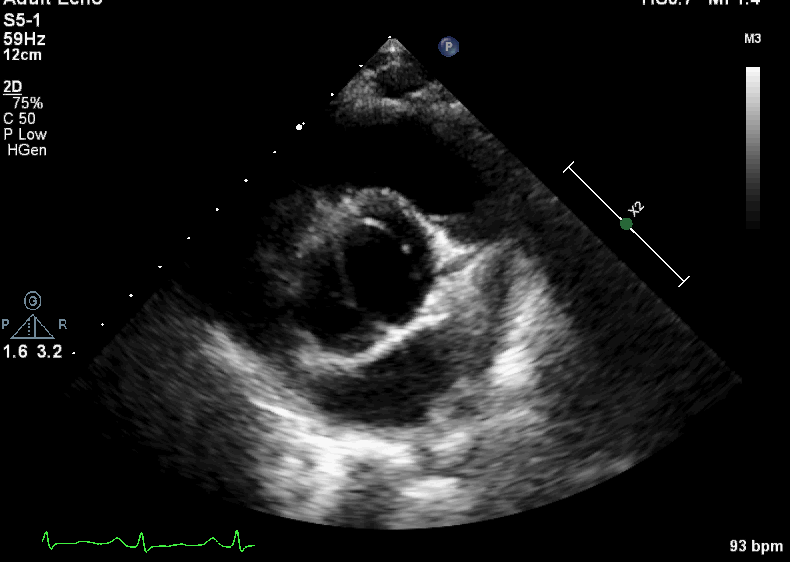

Supplement: Data S1 [file peerj-11-14950-s001.zip › Figure 1/BAV5.png]

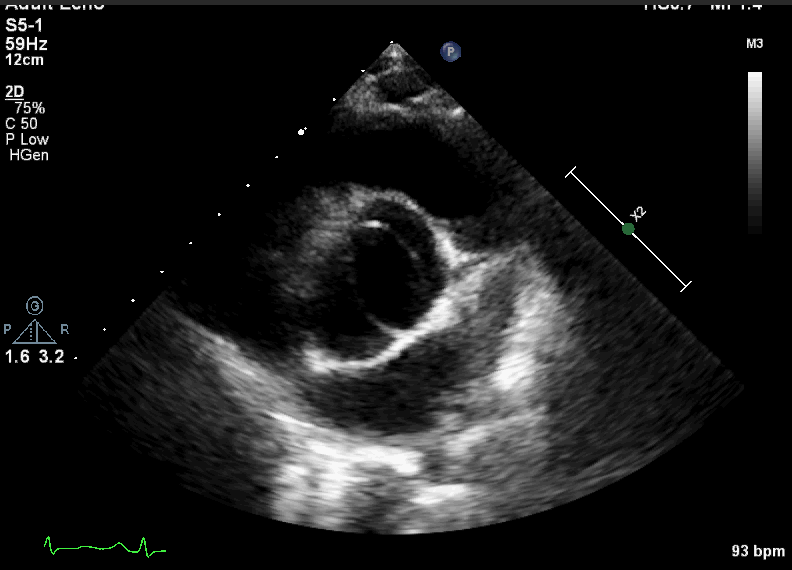

Supplement: Data S1 [file peerj-11-14950-s001.zip › Figure 1/BAV6.png]

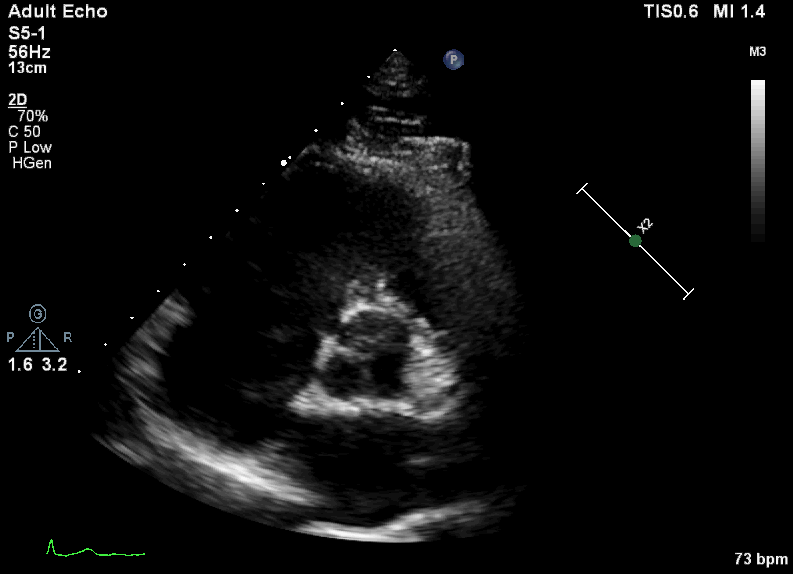

Supplement: Data S1 [file peerj-11-14950-s001.zip › Figure 1/TAV1.png]

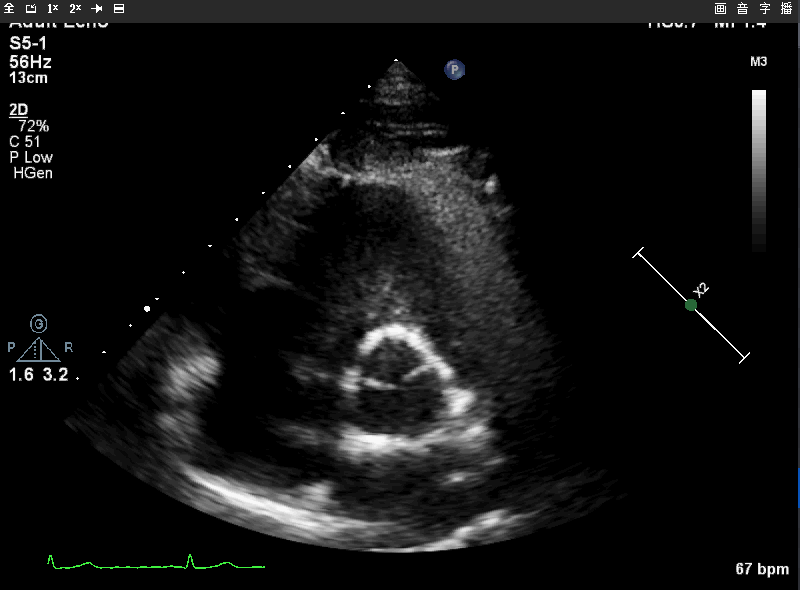

Supplement: Data S1 [file peerj-11-14950-s001.zip › Figure 1/TAV2.png]

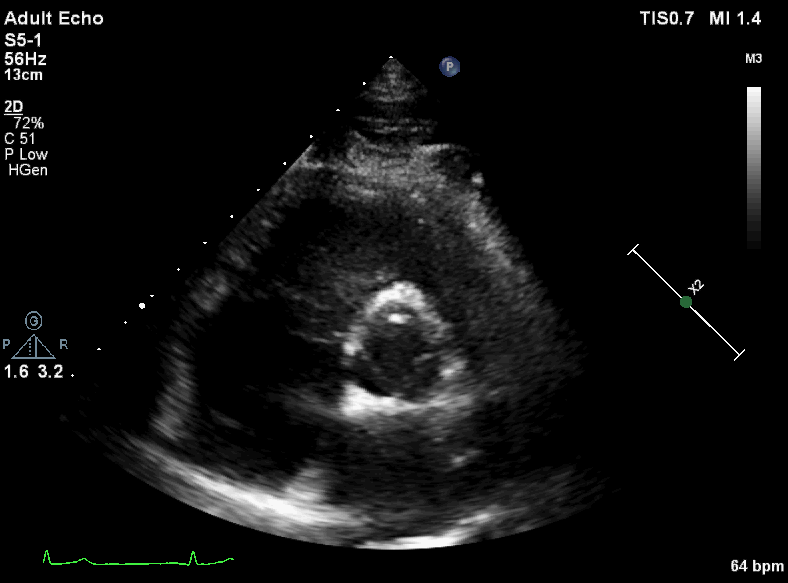

Supplement: Data S1 [file peerj-11-14950-s001.zip › Figure 1/TAV3.png]

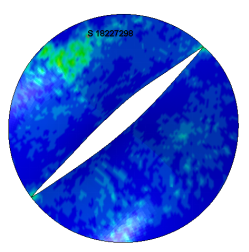

Supplement: Data S1 [file peerj-11-14950-s001.zip › Figure 3/25mmHg BAV close.tif]

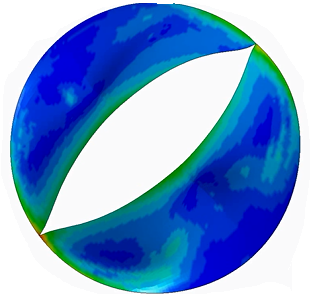

Supplement: Data S1 [file peerj-11-14950-s001.zip › Figure 3/25mmHg BAV open.tif]

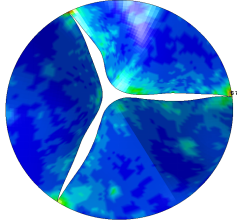

Supplement: Data S1 [file peerj-11-14950-s001.zip › Figure 3/25mmHg TAV close.tif]

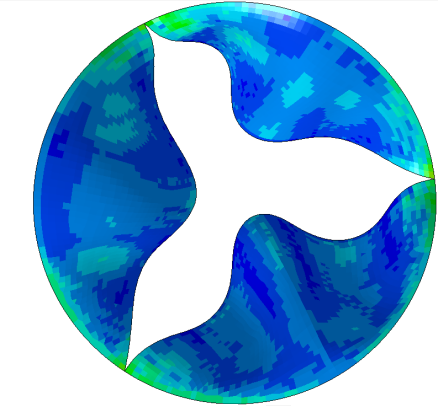

Supplement: Data S1 [file peerj-11-14950-s001.zip › Figure 3/25mmHg TAV open.tif]

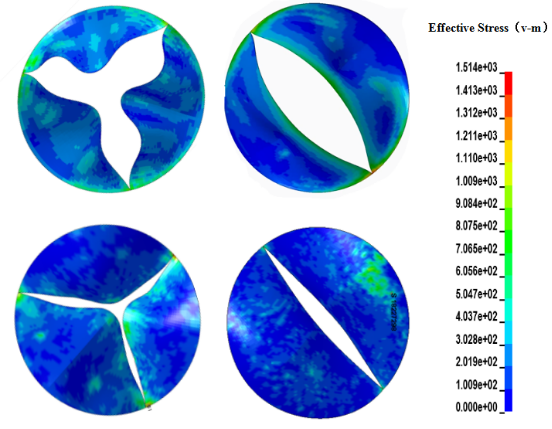

Supplement: Data S1 [file peerj-11-14950-s001.zip › Figure 3/25mmHg.tif]

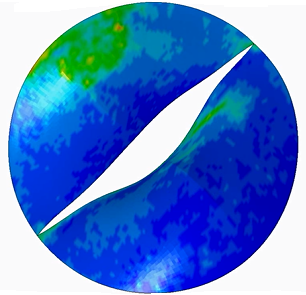

Supplement: Data S1 [file peerj-11-14950-s001.zip › Figure 4/50mmHg BAV close.tif]

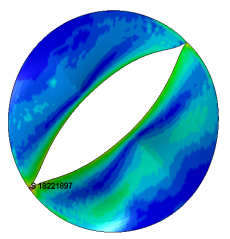

Supplement: Data S1 [file peerj-11-14950-s001.zip › Figure 4/50mmHg BAV open.tif]

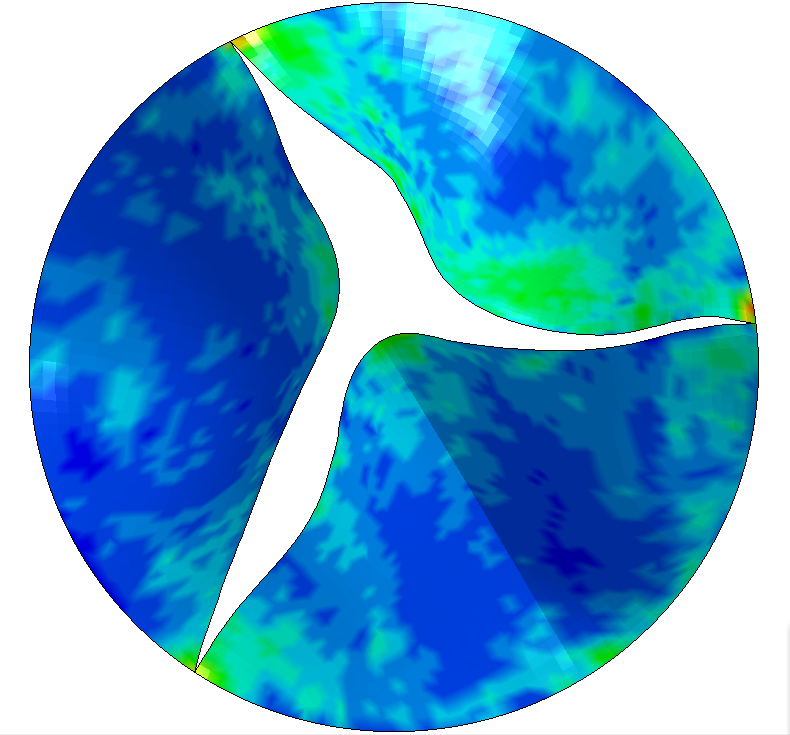

Supplement: Data S1 [file peerj-11-14950-s001.zip › Figure 4/50mmHg TAV close.tif]

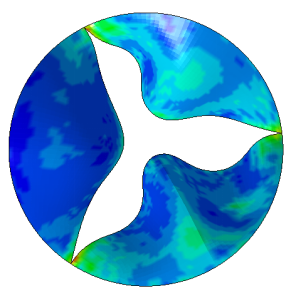

Supplement: Data S1 [file peerj-11-14950-s001.zip › Figure 4/50mmHg TAV open.tif]

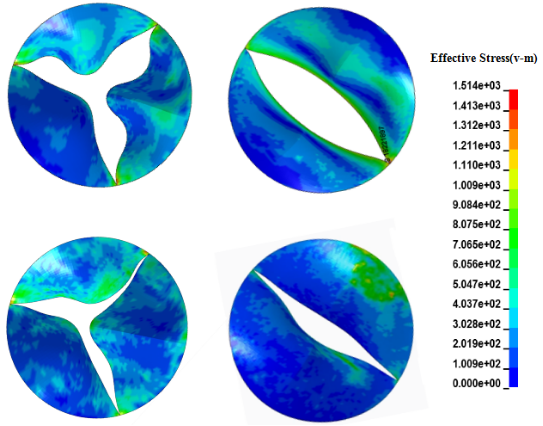

Supplement: Data S1 [file peerj-11-14950-s001.zip › Figure 4/50mmHg.tif]

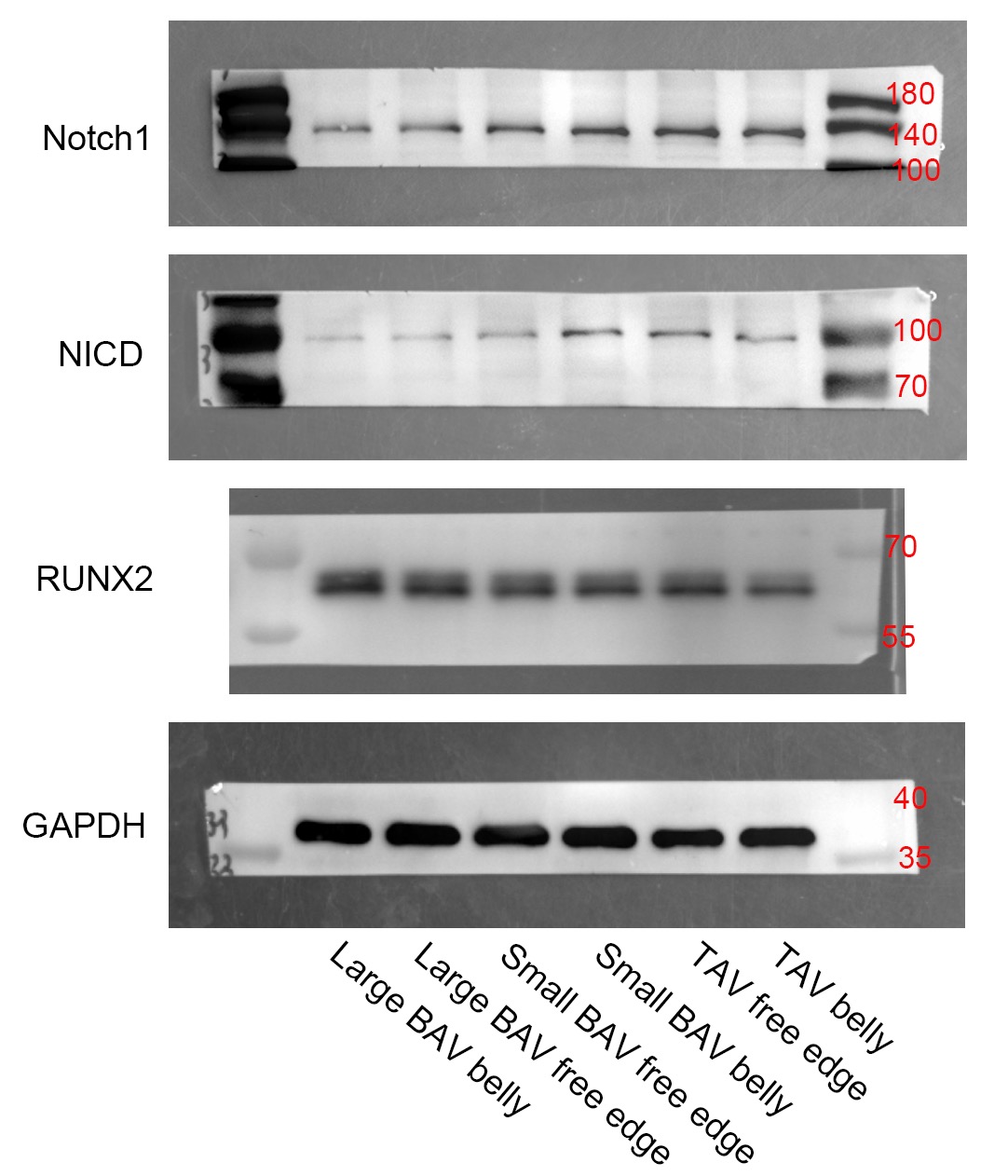

Supplement: Data S1 [file peerj-11-14950-s001.zip › Figure 5/Figure 5C.jpg]
